# MycoRRdb: A Database of Computationally Identified Regulatory Regions within Intergenic Sequences in Mycobacterial Genomes

**DOI:** 10.1371/journal.pone.0036094

**Published:** 2012-04-26

**Authors:** Mohit Midha, Nirmal K. Prasad, Vaibhav Vindal

**Affiliations:** Department of Biotechnology, School of Life Sciences, University of Hyderabad, Hyderabad, India; Université Paris-Sud, France

## Abstract

The identification of regulatory regions for a gene is an important step towards deciphering the gene regulation. Regulatory regions tend to be conserved under evolution that facilitates the application of comparative genomics to identify such regions. The present study is an attempt to make use of this attribute to identify regulatory regions in the *Mycobacterium species* followed by the development of a database, MycoRRdb. It consist the regulatory regions identified within the intergenic distances of 25 mycobacterial species. MycoRRdb allows to retrieve the identified intergenic regulatory elements in the mycobacterial genomes. In addition to the predicted motifs, it also allows user to retrieve the Reciprocal Best BLAST Hits across the mycobacterial genomes. It is a useful resource to understand the transcriptional regulatory mechanism of mycobacterial species. This database is first of its kind which specifically addresses *cis-*regulatory regions and also comprehensive to the mycobacterial species. Database URL: http://mycorrdb.uohbif.in.

## Introduction

Over the past few years the genomic sequence repertoire of mycobacterial sequences has increased tremendously. The availability of complete genome sequences makes it possible to efficiently employ computational approaches to understand the genome function and its complexity [1]. One of the important aspects to compare genome sequences is to find orthologous proteins among the existing species [2], [3]. The identification of orthologs is important not only to assist the functional annotation of a gene but also to identify its regulatory region. These regions are known to evolve at a slower rate than non-functional elements, and therefore finding the conserved DNA motifs within non coding region is an efficient method to predict these regions [4], [5]. Different approaches have been used to find the regulatory regions [6]–[8]. Generally, identification of these DNA elements relies on an extensive set of known target genes [4], [9]. Therefore, identification of regulatory region for a novel transcriptional regulator remains a challenging task.

Extensive research on mycobacteria has produced a number of online resources, providing information on pathogenicity, cellular physiology, operon arrangement, microarray, etc. [10]–[15]. These resources also include a database, MtbRegList, which contains the reported regulatory regions in *Mycobacterium tuberculosis* [16]. Nevertheless, there is still need to document the putative regulatory regions for all the mycobacterial genomes. Our present study addresses this issue, as it identifies the putative *cis-* regulatory sequences within the intergenic regions of mycobacterial species and also the similar DNA motif in a genome. In addition to the predicted regulatory regions, the database includes list of Reciprocal Best BLAST Hits (RBBHs) for all 25 mycobacterial species. The database also has a search feature to identify the sequences similar to a query DNA motif. This database can assist in the characterization of gene regulation in all the mycobacterial species.

## Methods

### Retrieval and filtering the genome sequences

The complete genome sequences of 25 *Mycobacterium species* were downloaded from NCBI ftp site (ftp://ftp.ncbi.nih.gov/genomes/Bacteria/). Some of the proteins were found to be present in more than one copy, identical in sequence, in certain species. In present study, such Multiple Identical Proteins (MIPs) were identified and replaced with only one representative protein sequence for further analysis.

### Identification of orthologs

Reciprocal Best BLAST Hit (RBBH) method was used to predict orthologous proteins in mycobacterial proteomes. Pairs of proteins, from two mycobacterial species, covering the at least 50% sequence length of both the proteins in alignment and E-values lower than of 10^−20^ for both directions using BLASTP program with all other parameters at default values were selected as RBBHs [17]–[19].

### Retrieval of operons and the intergenic sequences

Information for all mycobacterial operons in genomes of all 25 species were retrieved from the DOOR database (version2) [20], [21] Intergenic sequence upstream of the first gene of each operon was retrieved using perl script. Sets of intergenic sequences were compiled for each orthologous gene. These sets of sequences were further subjected to the identification of a regulatory region.

### Identification of regulatory regions

MEME suite was used to identify the conserved regulatory DNA elements from the set of sequences described earlier [4]. The DNA motif length, from minimum of 20 bases to maximum length of 30, was optimized using known DNA targets from *M. tuberculosis* [16]. DNA search was carried out to look for palindromes within the given strand as well as its complementary strand. Additionally, the top predicted DNA motifs observed associated with three or more orthologous sequences were selected as potential regulatory DNA element. All other parameters were kept on its default values. These DNA motifs were further searched in their respective genomes to identify the significantly similar motifs with minimum aligned length (L) of 16 bases (allowing N mismatch where N< = 0.2L; L-N>14).

### Database development

**Figure 1:**
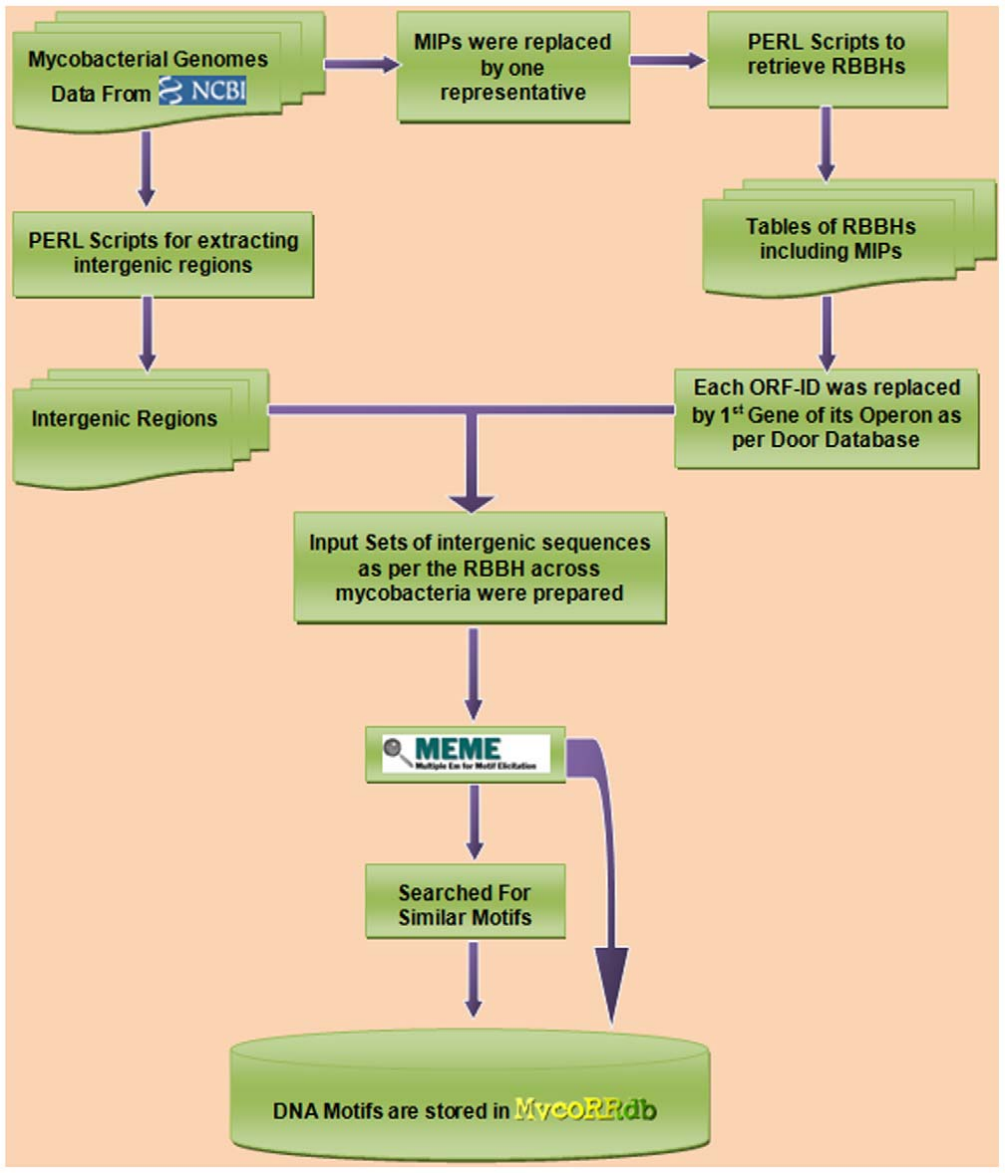
Flowchart of the methodology.

Subsequent to the identification of regulatory regions from all mycobacterial genomes, a web resource, MycoRRdb was developed. This database has been developed using MySQL. It is constructed to allow user to browse the outcome of study in an easy accessible mode. Web interface of the database is designed using PHP, HTML and Javascripts. Flow chart of the methodology followed in the study is depicted in Figure 1.

## Results and Discussion

### RBBHs across the mycobacterial species

The ortholog prediction is not only important to identify the regulatory region but also helps in functional annotation of a sequenced genome. Our study also began with the identification of the RBBHs which serves as potential ortholog. All the RBBHs from the mycobacterial species were identified using the methodology discussed. The identified lists of RBBHs for any mycobacterial gene across all 25 mycobacterial genome were used as a data source for the MycoRRdb.

### Mycobacterial regulatory regions

Subsequently, DNA regulatory regions were identified across the all 25 *Mycobacterium species*. The total predicted regulatory motifs were 37101 in number for all 25 mycobacterial genomes. Further, the motifs predicted across the Mycobacterial species were compared with the known DNA motifs reported in the literature [3], [5], [16], [22]–[41]. It was observed that 116 DNA motifs, out of 181 retrieved, were mapped in MycoRRdb and notified through the link given in the database. The comparative list of the predicted and the reported DNA motifs is given in Table S1. The maximum number of motifs was predicted from *Mycobacterium tuberculosis* H37Ra while the minimum number was from *Mycobacterium abscessus* ATCC 19977. These predicted DNA motifs are the putative Transcription Factor Binding Sites (TFBS). The TFBS identified, positioned at more than 400 nucleotide upstream to the translational start site, are highlighted with red colour font. Further in view of over representation, similar DNA motifs were searched to find the similar motifs within the predicted list of intergenic regulatory region. All the identified motifs are displayed with the strand information and the position from translational start site.

### Database access

**Figure 2:**
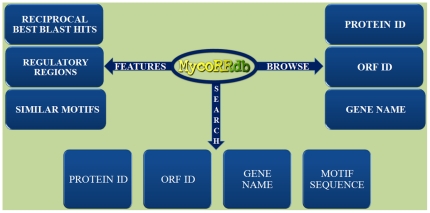
Schematic architecture of MycoRRdb.

**Figure 3:**
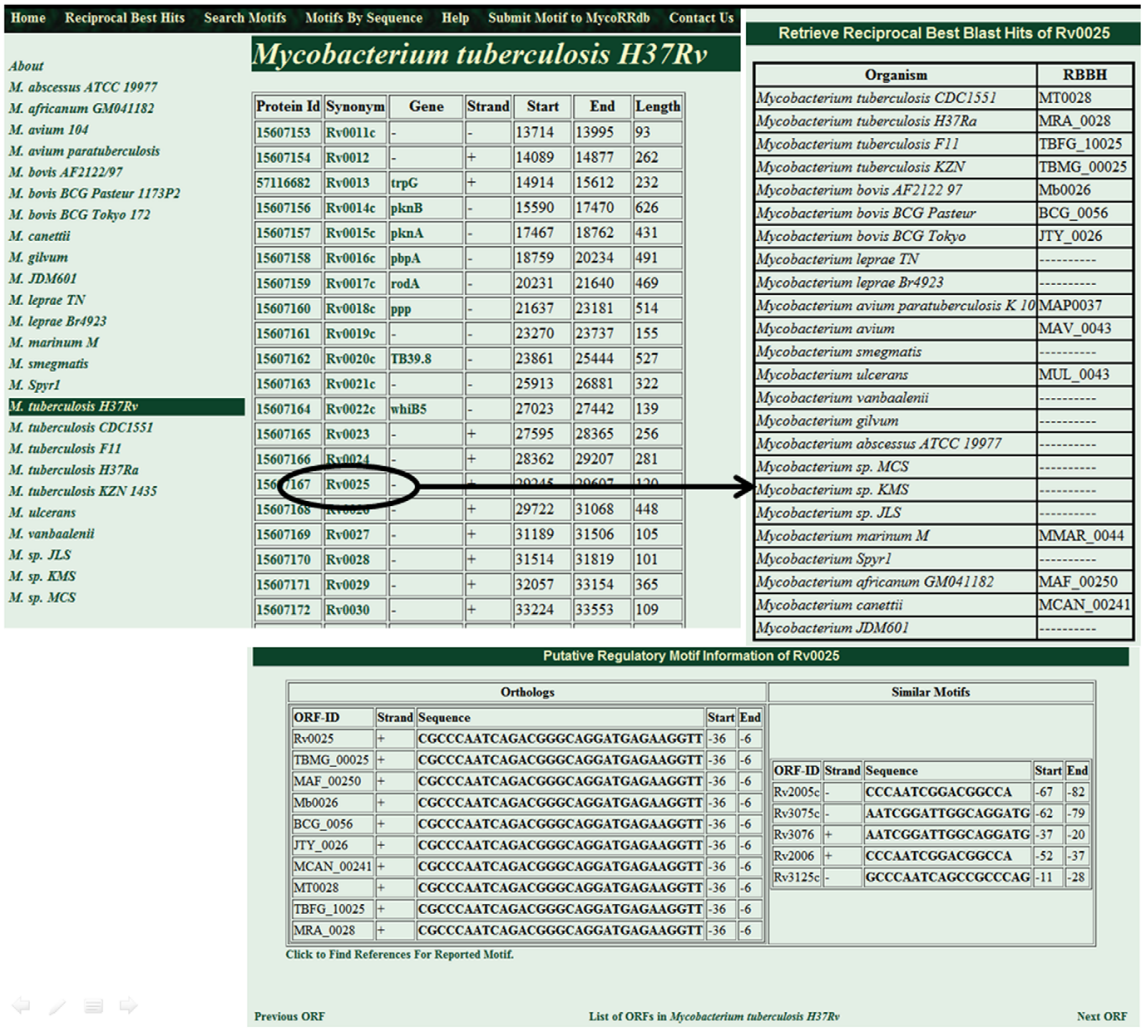
A browsable interface to retrieve RBBHS and DNA motifs.

MycoRRdb can be accessed through the database web interface at http://mycorrdb.uohbif.in. There are two kind of data that has been stored in MycoRRdb:(i)Reciprocal Best BLAST Hits (RBBHs), and (ii) Predicted Regulatory Region for each transcription unit (Figure 2). This information for any mycobacterial gene can be retrieved from MycoRRdb in either browsable or searchable fashion. Homepage of the database provides links for the mycobacterial genome which further leads to complete list of genes/protein id/ORF id of a particular species. From the list one can proceed to find the RBBHs of any gene across other mycobacterial species and associated regulatory DNA motifs along with its occurrence in the orthologous intergenic sequences. It also gives link, to facilitate user, to the retrieve the known motif reported in literature. In addition to this list of similar DNA motifs in a genome is also available (Figure 3).

**Figure 4:**
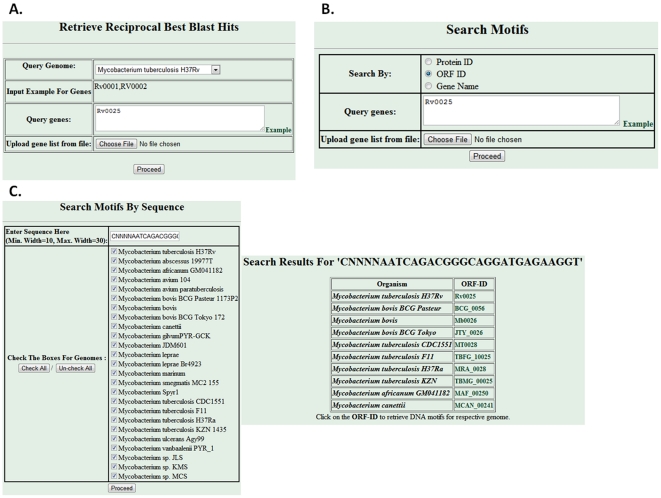
A searchable mode to retrieve RBBHS and DNA motifs. **A.** Interface to retrieve the RBBHs; **B.** Interface to retrieve the regulatory DNA motifs; **C.** Interface to retrieve the similar DNA motifs to the desired DNA sequence.

Besides browsing data from complete genes list, separate links have also been made available on web interface to quickly retrieve RBBHs or regulatory DNA motifs by gene name/protein id/ORF id. A searchable interface to retrieve RBBHs is shown in Figure 4A. The predicted regulatory regions and the similar sequences present in that genome can be also be retrieved by searchable interface using gene name/protein id/ORF id (Figure 4B). Moreover, user can scan the availability of its desired DNA sequence, if it exists in any Mycobacterial species, in the identified DNA motifs set of the Database (Figure 4C).

This database is under constant development to gather the experimentally validated DNA motifs to incorporate in the database. It also provides link for biologist to put forward the experimentally validated mycobacterial regulatory regions, if any.

### Conclusions

The availability of whole genome sequences makes Mycobacterium one of the highly sequenced genera. This wealth of sequence data provides unique opportunity to extract the genome information in order to address cellular physiology and to develop better intervention strategies for pathogenic species. This study is a systematic approach to reveal the putative regulatory regions and RBBHs across the mycobacterial species. On the one hand, the identified regulatory regions will help to understand the transcriptional regulation of the mycobacterial genes, and on the other hand, the identified RBBHs will assist to impart the functional knowledge of one gene to another. The availability of all the identified regulatory regions and RBBHs from the mycobacterial species at a websource, MycoRRdb, will help to access the data and will have potential implications to unravel the genomic complexity of the mycobacteria.

## Supporting Information

Table S1 DNA motifs in MycoRRdb mapped with regulatory regions reported in literature. (Available at: http://mycorrdb.uohbif.in/links.php).
(XLS)
